# Demographics and Fellowship Training of Residency Leadership in EM: A Descriptive Analysis

**DOI:** 10.5811/westjem.2016.10.31452

**Published:** 2016-11-21

**Authors:** Josh Greenstein, Ross Hardy, Jerel Chacko, Abbas Husain

**Affiliations:** * Staten Island University Hospital, Northwell Health, Department of Emergency Medicine, Staten Island, New York

## Abstract

**Introduction:**

Emergency medicine (EM) fellowships are becoming increasingly numerous, and there is a growing trend among EM residents to pursue postgraduate fellowship training. Scant data have been published on the prevalence of postgraduate training among emergency physicians. We aimed to describe the prevalence and regional variation of fellowships among EM residency leadership.

**Methods:**

We conducted an online anonymous survey that was sent to the Council of EM Residency Directors (CORD) membership in October 2014. The survey was a brief questionnaire, which inquired about fellowship, secondary board certification, gender, and length in a leadership position of each member of its residency leadership. We separated the responses to the survey into four different geographic regions. The geographic regions were defined by the same classification used by the National Resident Matching Program (NRMP). We defined residency leadership as program director (PD), associate PD and assistant PD. Residencies that did not complete the survey were then individually contacted to encourage completion. The survey was initially piloted for ease of use and understanding of the questions with a select few EM PDs.

**Results:**

We obtained responses from 145 of the 164 Accrediting Council for Graduate Medical Education-accredited EM residencies (88%). The fellowship prevalence among PDs, associate PDs, and assistant PDs was 21.4%, 20.3%, and 24.9% respectively. The most common fellowship completed was a fellowship in toxicology. Secondary board certification among PDs, associate PDs, and assistant PDs was 9.7%, 4.8%, and 2.9% respectively. Eighty-two percent of PDs have at least five years in residency leadership. Seventy-six percent of PDs were male, and there was a near-even split of gender among associate PDs and assistant PDs. The Western region had the highest percentage of fellowship and or secondary board certification among all levels of residency leadership.

**Conclusion:**

There is a low prevalence of fellowship training and secondary board certification among EM residency leadership, with the most common being toxicology. Assistant PDs, the majority of whom had less than five years residency leadership experience, had the highest percentage of fellowship training. There may be a regional variation in the percentage of residency leadership completing postgraduate training.

## INTRODUCTION

It is the perception that emergency medicine (EM) fellowships are becoming increasingly common due to a growing trend among EM residents to pursue postgraduate fellowship training. There are scant data on the prevalence of fellowships among EM physicians. We aimed to describe the prevalence and regional variation of fellowships among EM residency leadership. Additionally, completion of secondary board certification among EM residency leadership was also investigated, as this additional training is relevant to the primary study investigation.

## METHODS

We sent an online confidential survey to the Council of EM Residency Directors (CORD) membership list service in October 2014. The survey was closed on January 1, 2015. No incentives were offered. The institutional review board of our hospital approved this study. Individual emails were sent to each member of the CORD membership, with a link to the website of the survey (www.surveymonkey.com). The survey was a brief questionnaire, which inquired about fellowship training, secondary board certification, gender, and length of time in a leadership position of each member of its residency leadership. We defined residency leadership as program director (PD), associate PD and assistant PD. We did not define length of time in a leadership position, and this could have included a member who had completed a leadership role at a different institution as well. Lastly, we categorized responses into four different geographic regions as used by the National Resident Matching Program (NRMP) and Association of American Colleges (AAMC).^1^ Participants self-reported their residency affiliation and programs; we did not clarify which member of residency leadership responded - only which residency the responses were from. We also included responses from the researchers’ home institution. PDs of residencies who did not complete the survey were then individually contacted to encourage completion. This took place approximately six weeks after the initial survey request. Residency programs that still did not complete the survey were contacted one last time one month prior to completion of the study. The authors created the survey. A pilot survey was given to a consensus panel of three EM PDs to complete and comment on its clarity. We were then able to complete the survey without a need for any significant revisions to its content (Appendix). The survey ended with an open-ended question allowing for any additional responses or clarifications of the subject’s responses. We included responses only from residencies accredited by the Accrediting Council of Graduate Medical Education (N=164).

## RESULTS

We obtained responses from 145 of 164 residencies (88%). The table illustrates the breakdown of fellowship and secondary board certifications. The fellowship prevalence among PDs, associate PDs and assistant PDs was 21.4%, 20.3%, and 24.9% respectively. Secondary board certification prevalence among PDs, associate PDs, and assistant PDs was 9.7%, 4.8%, and 2.9% respectively. The most common fellowship completed was medical toxicology. The “other” category included various fellowships not listed in the survey. Examples of more common responses in the “other” category included but were not limited to fellowships such as global health and wilderness medicine. Some less common responses were a fellowship in sports medicine and cardiac emergencies. Internal medicine was the most common secondary board certification completed. Residency leaders who had completed a pediatric emergency medicine fellowship in addition to secondary board certification in pediatrics were only tallied as having completed a fellowship to ensure proper statistical analysis of the data.

The figure demonstrates the regional variation of residency leaders who had completed an EM residency and either a fellowship or secondary board certification. The four regions were Western, Northeastern, Central, and Southern. The Western region had the highest percentage of fellowship and secondary board certification across all levels of residency leadership. Greater than 46% of assistant PDs in the Western region had completed either a fellowship and/or secondary board certification.

## DISCUSSION

We found that there is an overall low prevalence of fellowship and secondary board certifications among residency leadership. The majority of PDs are male, with a near-even split among associate and assistant PDs. The Western region had the highest percentage of fellowship and secondary board certification among all levels of residency leadership. The results from this survey not only define the current demographics of fellowship and secondary board certification among EM residency leadership, but also suggest a growing trend of postgraduate training among residency leadership. PDs averaged the longest time in residency leadership and had the highest percentage of secondary board certifications. With regard to length of time in residency leadership position, no differentiation was made for whether this was at a single or multiple institutions. Assistant PDs who had the least experience had the highest prevalence of completed fellowships, and the least prevalence of secondary board certification. It is possible that there is a trend away from obtaining secondary board certifications and towards completion of fellowship training for those interested in obtaining a residency leadership position. This trend further varies by region. The Western region, considered by many to be a competitive academic EM job market, had the highest percentage of fellowship and secondary board certifications, as nearly 46% of assistant PDs have completed either a fellowship or secondary board certification. We speculate that the growing competitive job market for residency leadership positions may be, in addition to other variables, what drives residents to pursue postgraduate fellowship training. We recommend that emergency physicians contemplating a career in academic EM, and more specifically in residency leadership, should pursue additional training. We plan to repeat this study in 10 years, and it is our belief that the current trends will continue to reflect the changes already taking place.

Moreover, it would be interesting to study if the increased postgraduate training trend was exclusive to residency leadership or if there is an overall trend among all emergency physicians to pursue postgraduate training.

## LIMITATIONS

While survey-based studies are helpful in obtaining data and outlining trends, it is important to be aware that these studies are more vulnerable to subjectivity and interpretation. As with any survey, it is possible that responders may have interpreted some of the questions and answer choices differently. Even though we piloted the survey for ease of use and understanding, clarification was needed in one particular area. Many respondents included the American College of Emergency Physicians (ACEP) Teaching Fellowship, or Medical Education Research Certificate Program (MERC) as an education fellowship. We did not consider those programs to count as true fellowships as these are training workshops that do not involve the same amount of time investment as other EM fellowships. As a result, respondents were contacted to clarify their responses. We were able to track responses back to a specific residency program as the survey requested the program name. An additional limitation of the study is that the results obtained could be a reflection of the availabilities of fellowship at the time of residency leadership graduations. Many experienced faculty in EM residency leadership positions did not have fellowships available in their early careers, which may have led them to pursue secondary board certification. Lastly, even though there was an excellent response rate (88%), one could argue that the results might have been different with a higher percentage and might not truly describe additional education among EM residency leadership.

## CONCLUSION

There is an overall low prevalence of fellowship training and secondary board certification among EM residency leadership, with the most common being medical toxicology. However, the assistant PDs, who averaged the shortest length of time in leadership experience, had the highest percentage of additional fellowship training. This indicates a possible trend toward additional postgraduate training among residency leadership. There may be a regional variation with the Western region exhibiting the highest percentage of fellowship and secondary board certifications across all levels of residency leadership.

## Supplementary Information



## Figures and Tables

**Figure:**
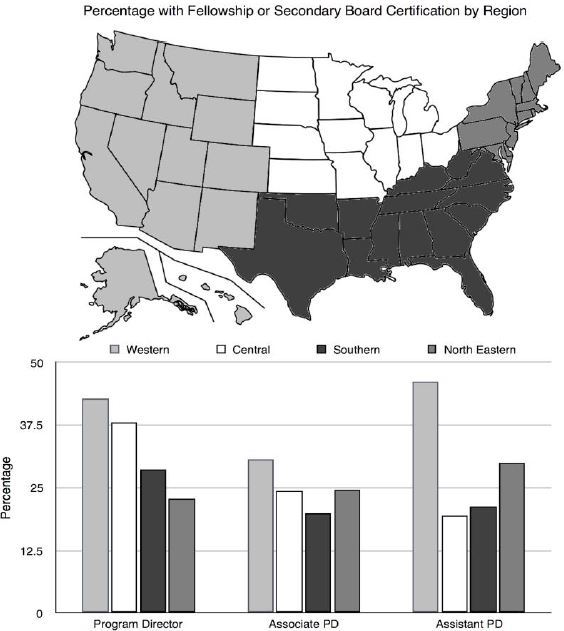
Combined fellowship and secondary board certification percentages of program directors, associate program directors, and assistant program directors, compared across geographic regions (Western, Central, Southern, and North Eastern as defined by the map).

**Table:** Fellowship, secondary board certifications, gender distribution, and length at leadership position of program directors, associate program directors, and assistant program directors.

	Program directors (N = 145)	Associate program directors (N = 187)	Assistant program directors (N = 177)
Fellowship			
None	114	149	149
Critical care	0	0	1(1%)
Education	7(5%)	2(1%)	8(5%)
EMS	2(1%)	3(2%)	0
Simulation	1(1%)	1(1%)	3(2%)
Pediatric	0	6(3%)	9(5%)
Ultrasound	3(2%)	8(4%)	9(5%)
Toxicology	8(6%)	7(4%)	6(3%)
Other	10(7%)	11(6%)	8(5%)
Second board certification			
None	131	178	172
Internal medicine	11(8%)	8(4%)	5(3%)
Pediatrics	0	0	0
Surgery	0	0	0
Other	3(2%)	1(1%)	0
Gender			
Male	110(76%)	105(56%)	96(54%)
Female	35(24%)	82(43%)	81(46%)
Leadership experience			
< 5 years	25(17%)	81(43%)	138(78%)
5–10 years	63(43%)	74(40%)	32(17%)
> 10 years	57(39%)	32(17%)	11(6%)

*EMS,* emergency medical services
