# Supplementary figures and images for: Demographics and Fellowship Training of Residency Leadership in EM: A Descriptive Analysis
Source: West J Emerg Med. 2016 Nov 21;18(1):129–32. doi: 10.5811/westjem.2016.10.31452 (PMC5226746; doi:10.5811/westjem.2016.10.31452)

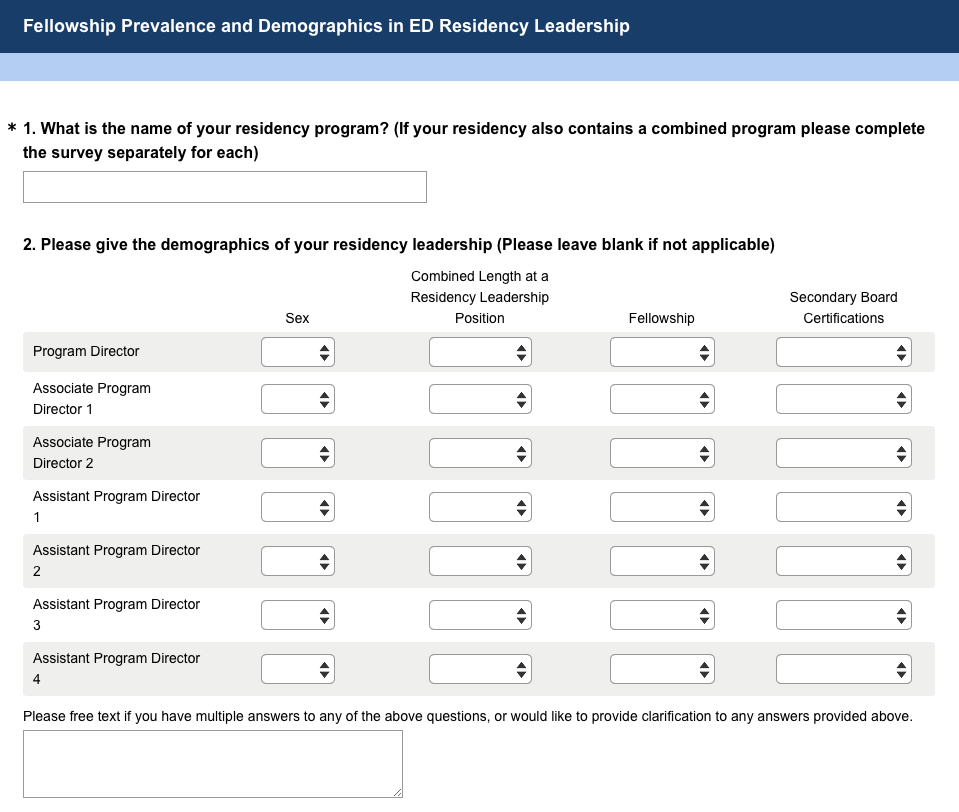

Supplement: Supplementary file 1 [file wjem-18-129-s001.docx]
